# Exercise during 14 days of head down tilt bedrest attenuates motor unit impairments in older humans

**DOI:** 10.1113/EP093398

**Published:** 2026-02-20

**Authors:** Philippe St‐Martin, Jean‐Christophe Lagacé, Mathil Ruel, Ahmed Ghachem, Guillaume Léonard, Eleonor Riesco, Mathew Piasecki, Jamie S. McPhee, Isabelle J. Dionne

**Affiliations:** ^1^ Faculty of Physical Activity Sciences Université de Sherbrooke Sherbrooke Quebec Canada; ^2^ Research Centre on Aging Université de Sherbrooke Sherbrooke Quebec Canada; ^3^ Institut Pluridisciplinaire Hubert Curien Université de Strasbourg, Centre National de la Recherche Scientifique Strasbourg France; ^4^ Faculty of Medicine and Health Sciences Université de Sherbrooke Sherbrooke Quebec Canada; ^5^ Centre of Metabolism, Ageing & Physiology (COMAP) Injury, Recovery & Inflammation Sciences, School of Medicine, NIHR Nottingham BRC University of Nottingham, Royal Derby Hospital Centre Derby UK; ^6^ Department of Sport and Exercise Sciences Manchester Metropolitan University Institute of Sport Manchester UK

**Keywords:** ageing, deconditioning, disuse, motor unit, spaceflight, strength

## Abstract

Head‐down tilt bedrest (HDBR) models the effects of mechanical unloading on neuromuscular function. The efficacy of exercise in preserving motor unit (MU) function in older adults during HDBR remains unclear. This study investigated the effects of 14‐day HDBR on MU properties in older adults and the protective role of exercise. Fifteen participants aged 55–65 years were randomized to Control group (*n* = 7, passive mobilization only) or Exercise group (*n* = 8, daily mixed resistance and aerobic training) during 14 days of strict HDBR. Knee extensor strength and leg lean mass were measured, and intramuscular EMG was used to record MU firing rate (MUFR), MU potential (MUP) area and complexity, and neuromuscular junction (NMJ) transmission instability during contractions normalized to 25% of pre‐bedrest strength. Plasma C‐terminal agrin fragment (CAF) was also measured. Following HDBR, knee extensor strength decreased more in the Control group (−33.4 N m; *P *< 0.001; ∼18% decrease) than in the Exercise group (−14.5 N m; *P *= 0.027; ∼8% decrease; interaction *P *= 0.045). Leg lean mass decreased similarly in both groups (−0.418 kg; *P *= 0.013). Exercise prevented the decrease in MUP area observed in Controls (−65 mV·ms, *P *= 0.240 vs. −253 mV·ms, *P* < 0.001) and led to a reduction in MUFR (−1.05 pulses/s, *P *< 0.001) not seen in Controls. NMJ transmission stability and CAF levels were unchanged in both groups. HDBR reduced leg lean mass and strength. Exercise attenuated declines in strength and MUP area, likely by preserving muscle fibre size despite reduced MUFR, without evidence of NMJ disruption. Exercise effectively attenuates neuromuscular decrements following HDBR in older adults, with implications for clinical care and spaceflight.

## Abstract (highlights)

- **What is the central question of this study?**
  What is the impact of 14 days of head‐down tilt bedrest on motor unit function in older adults, and can exercise attenuate these changes?
- **What is the main finding and its importance?**
  Bedrest reduced knee extensor strength and motor unit potential area, while exercise attenuated these declines. Neuromuscular junction transmission instability remained unchanged. These results demonstrate that exercise can protect neuromuscular health during short‐term disuse in older adults, informing strategies for clinical care and spaceflight.

## 1 INTRODUCTION

Periods of physical inactivity such as bed rest, limb immobilization and limb suspension are widely used in human studies to model the effects of mechanical unloading on skeletal muscle atrophy (Deane et al., 2024) and neuromuscular function (Piasecki, 2024), with applicability to medical recovery and spaceflight. Head‐down tilt bedrest (HDBR) is a well‐established model for simulating the effects of microgravity on the human body (Hajj‐Boutros et al., 2024). Unlike clinical observations in hospitalized patients, where the effects of bedrest are often confounded by the underlying pathology or pre‐existing inactivity preceding hospitalization, HDBR allows for the isolation of the physiological consequences induced by mechanical unloading per se in otherwise healthy older adults. This model has provided novel insights into the effects of spaceflight on multiple physiological systems and for developing effective countermeasures to mitigate these effects. It is now well established that with immobilization, strength declines at a greater rate than skeletal muscles atrophy (Hardy et al., 2022; Marusic et al., 2021), which has prompted greater focus on the neural contributions to strength losses.

Muscle force production is regulated by the recruitment of motor units (MU) and the rate at which they discharge (MU firing rate; MUFR) (Enoka & Duchateau, 2017). In younger adults, vastus lateralis MUFR is reduced after unilateral limb immobilization (Inns et al., 2022) and suspension (Sarto et al., 2022), particularly for the lower threshold MUs which likely contributes to the reduction of maximal strength (Piasecki, 2024; Valli et al., 2023). However, reduced discharge rates may not solely represent a neural deficit. It has been proposed that this reduction may also reflect a physiological adjustment, often termed ‘muscle wisdom’, whereby the nervous system downregulates firing rates to optimize force summation in response to the slowed muscle contractile kinetics often observed with disuse (Ruggiero & Gruber, 2024). Molecular alterations, including novel biomarkers of neuromuscular junction (NMJ) remodelling such as the C‐terminal agrin fragment (CAF), have been reported after 10 days of bed rest (Monti et al., 2021) and 10 days of unilateral limb suspension (Sarto et al., 2022) in young adults. Similar effects were also noted in older men following 10 days of bedrest, coinciding with electrophysiological markers of NMJ dysfunction (Motanova et al., 2025). These molecular and structural changes appear to precede functional changes affecting the neuromuscular transmission stability. Importantly, these alterations are reversible with exercise rehabilitation (Sarto et al., 2022), highlighting the strong restorative potential of physical exercise after disuse.

Substantial evidence indicates that exercise countermeasures implemented during periods of disuse can partially or even fully attenuate the loss of skeletal muscle mass and strength in young adults (X. Guo et al., 2025). However, it remains unclear whether these interventions are equally effective in preserving MU and NMJ function, particularly in older adults. This gap in knowledge is notable given that older individuals are generally more prone to inactivity (Gomes et al., 2017; Hawkins et al., 2009; Lee et al., 2025; Murtagh et al., 2015; Troiano et al., 2008) and exhibit greater susceptibility to disuse‐related impairments (Nunes et al., 2022; Reidy et al., 2018; Suetta et al., 2009). Understanding the physiological adaptations of MUs in response to disuse in ageing populations is critical, as disuse‐induced weakness and atrophy are likely to exacerbate the progressive neuromuscular declines associated with ageing and contribute to the development of sarcopenia. Furthermore, age‐related changes in MU structure and function may render older adults more vulnerable to the detrimental effects of disuse (Y. Guo et al., 2024; Piasecki et al., 2018; Piasecki, Ireland, Jones et al., 2016).

Electromyography (EMG) provides a powerful tool for assessing neuromuscular function, offering insights into these physiological adaptations. For instance, surface EMG techniques that decompose signals to isolate individual MU activity during voluntary contractions are now widely applied in human research (Del Vecchio et al., 2020). However, their use is limited in deeper muscles, where signal detection is attenuated by overlying subcutaneous tissue (Besomi et al., 2019; Kuiken et al., 2003). In such cases, intramuscular EMG (iEMG) provides a well‐established alternative, allowing direct sampling of MU spike trains at multiple depths (Jones et al., 2021) and offering the additional advantage of recordings closer to the site of origin, thereby enabling assessment of parameters relevant to NMJ transmission (Piasecki et al., 2021; Stålberg & Sonoo, 1994).

While disuse‐induced declines in muscle mass, strength and neuromuscular function are well characterized in younger adults, relatively few studies have directly examined these phenomena in older individuals or assessed MU and NMJ outcomes in this age group. For example, a systematic review indicates that older adults experience more rapid losses of leg lean mass and leg strength during bed rest compared with younger adults (Di Girolamo et al., 2021). A recent 10‐day horizontal bed rest study in older men further demonstrated reductions in MUFR, increased complexity of MU potentials (MUPs), evidences of NMJ dysfunction, and elevated circulating CAF concentrations, collectively reflecting ongoing NMJ remodelling (Motanova et al., 2025). However, to date, no studies have investigated HDBR in older adults or evaluated whether a combined exercise countermeasure during disuse can preserve MU firing behaviour and NMJ transmission in this population.

The present study investigated the effects of 14‐day HDBR on neuromuscular function in males and females aged 55–65 years and evaluated whether a mixed exercise countermeasure could mitigate these effects compared to a control condition. We hypothesized that HDBR would reduce MUFR and increase neuromuscular transmission instability, and that the exercise intervention would attenuate these adaptations.

## 2 METHODS

### 2.1 Ethical approval

All participants received both verbal and written information about the study before providing written informed consent. The study was conducted in accordance with the *Declaration of Helsinki* and approved by the ethical committees of the McGill University Health Centre (MP‐37‐2021‐7170) and the CIUSSS de l'Estrie–CHUS (no. 2021‐7170). The Bedrest Study in Older Adults (BROA) is also registered in the Clinical Trials Database under the identifier NCT04964999.

### 2.2 Experimental overview

This study is part of the BROA project (the first Canadian HDBR multicentre study; a two‐arm randomized control trial conducted in Montreal, Canada) (Hajj‐Boutros et al., 2024). Funded by the Canadian Institutes of Health Research, the Canadian Space Agency, and the Canadian Frailty Network, the study recruited 24 participants from the greater Montreal area. Once eligibility was confirmed by phone (requirements for inclusion and exclusion criteria, psychological, diet and physical activity investigations), participants were invited to the laboratory for a screening visit. Inclusion and exclusion criteria were harmonized across all teams to ensure safety and data integrity for all experimental modules (Hajj‐Boutros et al., 2024). Participants were excluded if they demonstrated abnormalities on electrocardiograms (ECG), had a history of HIV, hepatitis B, anaemia, as defined by sex‐specific haemoglobin or ferritin levels below established reference ranges, or a family history of thrombosis. Participants with bone mineral density measurements by dual‐energy X‐ray absorptiometry (DXA) that were greater than 2 standard deviations from age‐ and sex‐matched reference values (i.e., a T‐score exceeding ±2) were excluded from the study. Participants who reported a predominantly sedentary lifestyle, defined as sitting for more than 7 h per day, were not eligible for inclusion. Those adhering to special diets, including vegetarian, vegan or other restrictive dietary practices, were also excluded. Individuals with subcutaneous or implanted medical devices, such as pacemakers, intracardiac devices, infusion pumps, aneurysm clips, dental implants or tissue expanders, were not permitted to participate. Participants who had donated blood within 3 months preceding the start of the study were excluded. Smoking tobacco or marijuana mixed with tobacco (containing tetrahydrocannabinol, THC) within 6 months prior to enrolment precluded participation. Individuals with a reported history of drug abuse were excluded. Participants who consumed excessive alcohol (defined as more than 10 drinks per week or more than two drinks per day on most days) or who used illicit or controlled substances (e.g., THC, cocaine, opiates, amphetamines/methamphetamines, benzodiazepines, barbiturates, buprenorphine, methadone metabolites, oxycodone or phencyclidine) within 30 days prior to study initiation were not eligible. Use of ergogenic supplements such as creatine during the same period was also an exclusion criterion. Additionally, individuals who had participated in another research study within 2 months prior to this study were excluded. Finally, participants who tested positive for COVID‐19 within the week preceding the start of the study were excluded from enrolment. Participants attended baseline visits on day 0, were ambulatory for the initial 5 days, then maintained in a microgravity analogue bedrest (head down bedrest; HDBR) continuously for 14 days. Post assessments were made immediately following the 14‐day HDBR. Following HDBR, participants recovered for 7 days in the laboratory while being ambulatory.

During HDBR, participants were randomly divided into two groups, Control and Exercise. In line with standard HDBR protocols, all participants underwent daily passive physical therapy sessions conducted by a certified physical therapist. These sessions consisted of stretching, joint mobilization and massage aimed at alleviating discomfort and preventing venous thrombosis. In addition, participants in the Exercise group performed a mixed countermeasure programme consisting of 1 h per day of resistance and aerobic training. The rationale and details behind the standardized multimodal countermeasure are described elsewhere (Hajj‐Boutros et al., 2024; Hedge et al., 2022). Briefly, the intervention comprised three sessions per day alternated between aerobic and resistance training. Aerobic exercise involved three approaches: (1) high‐intensity interval training performed on a supine cycle ergometer, (2) continuous aerobic training on the cycle ergometer for either 15 or 30 min, and (3) progressive aerobic training. Resistance training included two weekly sessions for the lower body and two for the upper body, using cables, resistance bands and body weight, all performed while participants remained in bed, in a head‐down tilt or horizontal position. All training sessions were supervised and tailored by a certified exercise physiologist.

Twenty‐three older adults completed the 14‐day HDBR campaign as part of the larger Canadian Bedrest Study (Hajj‐Boutros et al., 2023). All 23 participants underwent the neuromuscular assessments described below. However, due to the strict technical requirements for intramuscular EMG decomposition (e.g., signal stability, absence of interference), only 15 participants provided complete, analysable datasets across all time points. Therefore, the final analytical sample for this study consisted of 15 participants.

### 2.3 Muscle mass and strength

Participant lower limb lean mass was measured by DXA scan (Lunar Progidy DXA, GE Healthcare, Madison, WI, USA). Participants remained in the scanner for about 30 min while two X‐ray beams with varying energy levels measure the major body compartments, including bone mineral and soft tissue, which is further subdivided into fat and lean body mass. Prior to the strength assessments, participants were instructed on the equipment and proper exercise technique. A quantitative multi‐joint muscle Biodex dynamometer (Biodex System 3, Mirion Technologies Inc., Florham Park, NJ, USA) was used to assess maximal voluntary strength of the knee‐extensor muscles on the right leg. In an upright seated position, with the hips and knees flexed at 90°, the participant was asked to perform three maximal voluntary isometric contractions (MVCs), each lasting 5 s, at a 90° angle, with 1 min of rest between attempts. Belts were secured at the hips and over the trunk to prevent the use of other muscle groups. Standardized verbal encouragement was provided throughout the test, without visual feedback to ensure maximum contraction, regardless of the results of the previous attempt.

### 2.4 Intramuscular EMG

Participants were seated upright in a customized isometric dynamometer chosen to minimize electromagnetic interference for optimal signal quality. The right ankle was positioned in a neutral position, with both knee and hip angles maintained at 90°. The hips were secured to limit extraneous movements, and the ankle was securely fastened to the dynamometer with inextensible straps. The central motor point of the vastus lateralis was identified using low‐intensity percutaneous electrical stimulation, as previously described (Sarto et al., 2022). The skin around the vastus lateralis muscle belly was cleaned with alcohol swabs.

iEMG signals were recorded using a 26‐gauge disposable concentric needle electrode (TECA, S53155 or S53156, Natus Medical Inc., Pleasanton, CA, USA) inserted diagonally at approximately 80° to approximately 1.5 cm deep into the muscle belly, at the motor point (Sarto et al., 2022). Participants performed a series of four to six submaximal voluntary contractions, each lasting 20 s with 45 s of rest, at a target intensity of 25% of the peak MVC torque previously determined on the Biodex dynamometer. This fixed absolute target was chosen to assess the neural adjustments required to maintain a constant force output following the intervention. Between each contraction, the concentric needle position was either rotated by 180° or withdrawn approximately 3 mm to record from MU in spatially distinct regions (Jones et al., 2021). During the test, participants were provided with real‐time audio feedback of their torque by an investigator to help them to maintain a stable contraction. iEMG signals were band‐pass filtered at 10 Hz to 10 kHz, recorded at 40 kHz and amplified/digitized/recorded (LabChart, ADInstruments, Sydney, Australia). All post‐intervention MUPs were recorded at contraction levels normalized to 25% of the pre‐bedrest MVC.

MUP trains (MUPTs) were acquired using the same procedures previously described (Piasecki, Ireland, Coulson et al., 2016). Briefly, decomposition‐based quantitative electromyography (DQEMG) software was used to detect MUP and their corresponding MUPTs. All MUPs were visually inspected, and parameters adjusted where necessary; MUP area was taken as the total area within the MUP duration (onset to end) and is indicative of MU size. The number of turns is a measure of MUP complexity and classified as a change in waveform direction of at least 25 µV, which indicates the level of temporal dispersion across individual muscle fibre contributions to a single MUP. MUFR was assessed as the rate of MUP occurrences within a MUPT, expressed as the number of occurrences per second (Hz). MUFR variability is reported as the coefficient of variation (CV) for the interspike interval, displayed as a percentage. Near‐fibre (NF) jiggle is a measure of the shape variability of consecutive near‐fibre MUPs of an MUPT, expressed as a percentage of the total NF MUP area (Piasecki et al., 2021). MUP negative peak slope ratio was calculated as the absolute value of the rise of the MUP template negative peak, across the 500 µs interval before the negative peak, divided by the fall of MUP template negative peak, across the 500 µs interval after the peak, and represents relative rates of ion exchange during the depolarization and repolarization phases of an action potential (Jones et al., 2023). Editing of the MUPs/MUPTs was agreed by two evaluators (P.StM. and J.S.M.) and the results were confirmed independently by MP.

### 2.5 CAF

A commercially available enzyme‐linked immunoassay (ELISA) kit was used to assess plasma CAF concentrations (ab216945, Abcam, Cambridge, UK). This assay has a measurement range of 28.13 pg/ml to 1800 pg/ml. Plasma was only thawed once, and all samples and standards were run in duplicate. CAF concentrations in the samples were interpolated from their respective standard curves and corrected for dilution. The coefficients of variation (CV) were 2.4% for intra‐assay and 4.5% for inter‐assay measurements.

### 2.6 Statistical analysis

All statistical analyses were performed in RStudio (version 2022.12.0 for Windows). Participant characteristics are presented as means ± standard deviation (SD) and median [Interquartile range (IQR)] in Table 1. Multilevel mixed‐effects linear regression models were fitted using the lme4 package in R (Bates et al., 2015), with Time (bedrest effect) and Condition (group effect) included as fixed effects, along with their interaction (Time *×* Condition). Where significant interactions were detected, estimated marginal means (EMMs) and 95% confidence interval (CI) were calculated using the EMMs package to explore pairwise differences. MU data are presented as individual MUs, participant means and group EMMs from each model output. Statistical significance was accepted when *P* < 0.05.

**TABLE 1:** Baseline participant characteristics.

	Control (*n* = 7; 4M, 3F)		Exercise (*n* = 8; 3M, 5F)	
Characteristic	Means ± SD	Median [IQR]	Means ± SD	Median [IQR]
**Age (years)**	58.6 ± 3.1	58.0 [3.5]	60.0 ± 2.6	60.0 [3.75]
**Height (cm)**	168.6 ± 13.3	165.4 [9.4]	166.2 ± 8.2	164.5 [11.1]
**Weight (kg)**	70.2 ± 17.8	73.4 [17.9]	70.7 ± 14.6	72.4 [23.9]
**BMI (kg/m^2^)**	24.4 ± 3.1	25.5 [4.0]	25.4 ± 3.4	26.2 [5.6]

Data are presented as means ± SD and median [IQR]. IQR, interquartile range; SD, standard deviation.

## 3 RESULTS

A total of 15 older adults completed the necessary assessments within the HDBR trial, consisting of seven in the Control group (4 males, 3 females) and eight in the Exercise group (3 males, 5 females). Participant baseline characteristics of the 15 included participants are presented in Table 1 and did not differ between groups.

**FIGURE 1:**
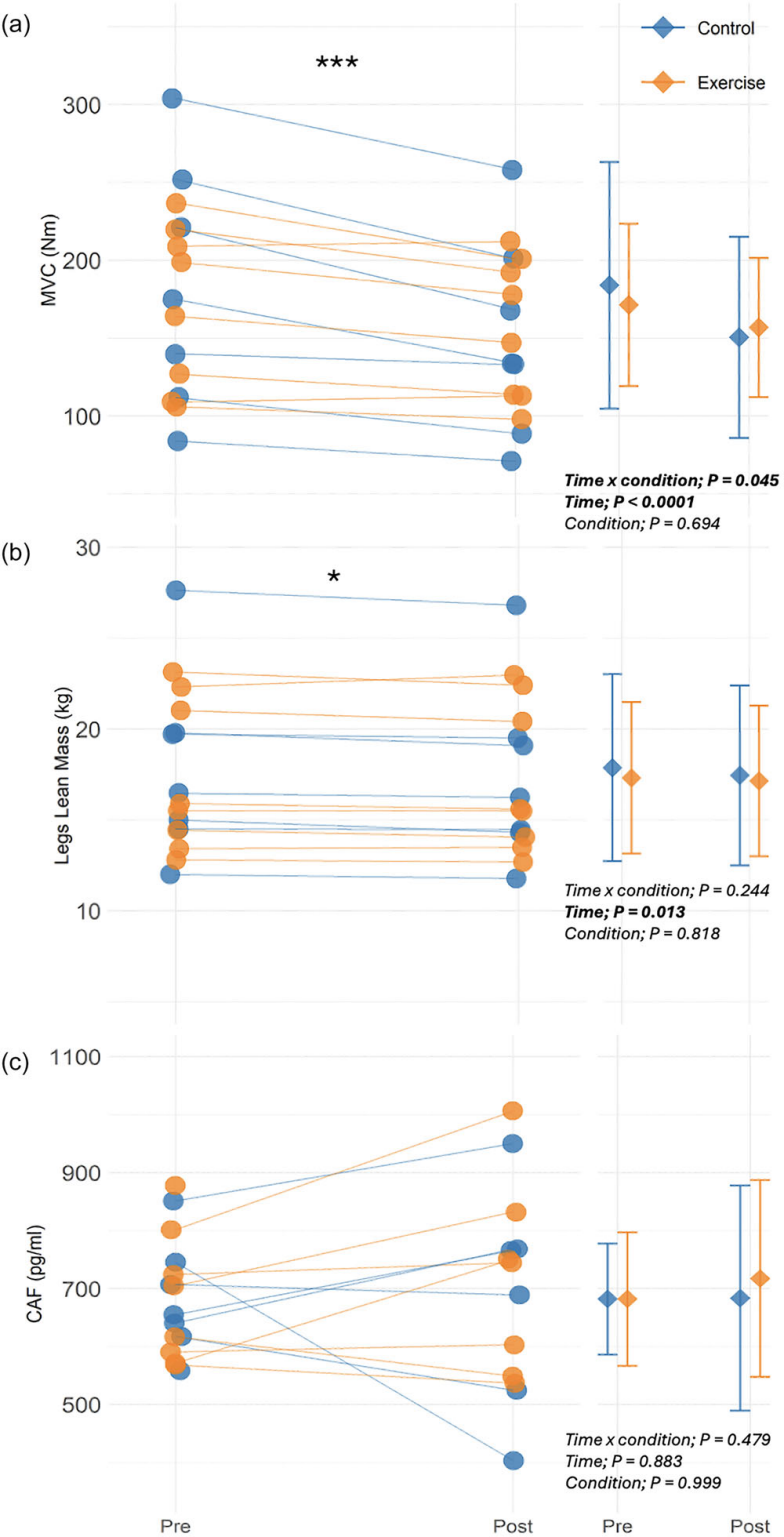
Knee extensor maximal voluntary contraction (a), leg lean mass (b), and plasma CAF concentrations (c) in Control (*n* = 7; blue) and Exercise (*n* = 8; orange) groups. Left panel shows individual values pre and post, and right panel shows group mean and standard deviation. *P < 0.05, ***P < 0.001. CAF, plasma C‐terminal agrin fragments; MVC, maximum voluntary contraction.

There was a statistically significant Time × Condition interaction for MVC (*P = *0.045), along with a main effect of Time (*P *< 0.001), but no effect of Condition (*P *= 0.694). Pairwise comparisons showed EMMs of muscle strength decreased by 33.4 N m (95% CI: 20.0 to 46.9; *P *< 0.001) from pre‐ to post‐intervention in the Control group, and 14.5 N m (95% CI: 1.94 to 27.1; *P *= 0.027) in the Exercise group (Figure 1a).

For DXA‐derived leg lean mass, there was no significant Time *×* Condition interaction (*P *= 0.244), nor a main effect of Condition (*P *= 0.818). However, a significant main effect of Time was observed, corresponding to a mean decrease of −0.418 kg from pre‐ to post‐bed rest (95% CI: −0.733 to −0.102; *P *= 0.013). Plasma CAF concentrations showed no significant Time *×* Condition interaction (*P *= 0.479), nor main effects of Time (*P *= 0.883) or Condition (*P *= 0.999) (Figure 1c).

### 3.1 MU parameters

**FIGURE 2:**
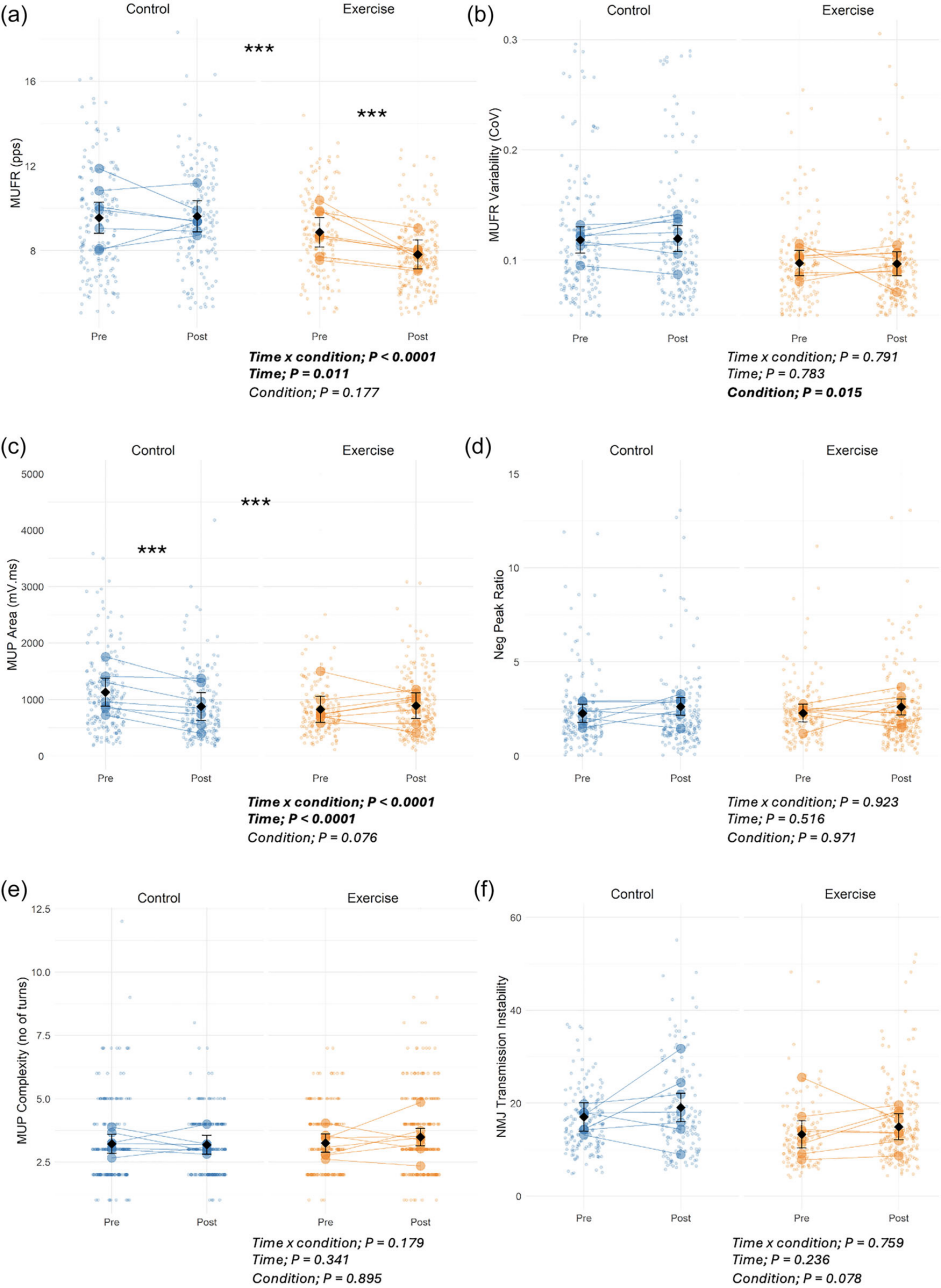
Individual MU parameters pre‐ and post‐bedrest in Control (blue) and Exercise (orange) groups. Small dots show individual MUs (369 MUPs pre‐bedrest: Control, 189; Exercise, 171; and 454 MUPs post‐bedrest: Control, 197; Exercise, 257), larger circles indicate participants means, linked from pre to post, and black diamonds and error bars indicate EMMs with 95% CI from each model. Centred asterisks indicate significant interaction, within group asterisks indicate within group EMMs; ***P < 0.001. CoV, coefficient of variation; MUP, motor unit potential; NMJ, neuromuscular junction; pps, pulses/s.

A total of 814 MUPs and corresponding MUPTs were recorded, comprising 369 MUPs prior to bedrest (pre: Control, 189; Exercise, 171) and 454 MUPs following bedrest (post: Control, 197; Exercise, 257). For MUFR there was a significant Time × Condition interaction (*P *< 0.001), a significant effect of Time (*P *= 0.011), and no effect of Condition (*P *= 0.177). EMMs showed a mean decrease of 1.05 (95% CI: −1.46 to −0.65; *P *< 0.001) pulses/s in the Exercise group with no change in the Controls (*mean change*: −0.07 pulses/s; 95% CI: −0.49 to 0.34 *P *= 0.733; Figure 2a). For MUFR variability, there was no Time × Condition interaction (*P *= 0.791), or effect of Time (*P *= 0.783), but there was an effect of Condition (*P *= 0.015), with a lower MUFR variability in the Exercise group compared to Controls (Figure 2b).

There was a significant Time × Condition interaction for MUP area (*P *< 0.001), a main effect of Time (*P *< 0.001), but no significant effect of Condition (*P *= 0.076). EMMs showed a significant decrease of 253 mV·ms (95% CI: −364 to 142; *P *< 0.001) in the Control group from pre to post, with no change in the Exercise group (*mean changes*: 65 mV·ms; 95% CI: −44 to 174; *P *= 0.240; Figure 2c). There was no significant Time × Condition interaction, and no main effect of Time or Condition on MUP negative peak ratio (all *P *> 0.5; Figure 2d). MUP complexity, quantified as the number of MUP turns, did not differ with Time or Condition, nor was there a significant Time × Condition interaction (all *P *> 0.17; Figure 2e). There was no change in NMJ transmission instability following the intervention, with no effect of Condition, nor a Time × Condition interaction (all *P *> 0.1) (Figure 2f).

## 4 DISCUSSION

This study used iEMG to investigate the impact of 14 days of HDBR on neuromuscular function and MU properties in older males and females. To the best of our knowledge, this study is the first to assess the efficacy of exercise countermeasures to attenuate the anticipated adverse effects. Although both groups exhibited reductions in knee extensor strength and leg lean mass with HDBR, the greater declines in strength observed in the Control group suggest that exercise performed during bedrest significantly mitigated the effects of disuse. This beneficial effect of exercise countermeasures extended to MU characteristics with the Exercise group showing a reduction in MU discharge rates at standardized force levels, whereas the Control group exhibited a decrease in MUP area. We also found that NMJ transmission instability and circulating plasma CAF concentrations remained unchanged in both groups after HDBR, indicating limited NMJ adaptation over the bedrest period.

The observation that muscle strength and mass declined in both groups aligns with previous reports (Inns et al., 2022; Marusic et al., 2021; Sarto et al., 2022). However, our study goes further by providing new insights into the effects of exercise, which minimized the loss of strength following bedrest in older adults. We also provide novel insight into the MU changes in older adults and the effectiveness of exercise countermeasures. At forces normalized to 25% MVC pre‐intervention, MUP area significantly decreased in the Control group, whereas it was preserved in the Exercise group. The size of a needle‐recorded MUP is strongly influenced by both the number and size of individual muscle fibres closest to the electrode (Piasecki et al., 2021; Stålberg et al., 2019). Thus, the observed difference could suggest a relative decline in fibre number or fibre cross section in the Control group, but a preservation of these properties in the Exercise group. While our DEXA analysis of total leg lean mass did not detect a significant interaction, this interpretation is supported by volumetric muscle analysis from the same cohort (Dulac et al., 2024). Dulac and colleagues reported a significant decrease in upper quadriceps volume in the Control group following HDBR, whereas volume was preserved in the Exercise group. This anatomical confirmation suggests that the reduction in MUP area observed in Controls likely reflects genuine structural remodelling within the MU, which was successfully mitigated by the exercise countermeasure. Disentangling the contributions of fibre number or fibre cross sectional area changes to these observed effects is experimentally challenging, but changes to both are plausible, with evidence from human bedrest studies showing denervation (Motanova et al., 2025) and fibre atrophy (Dirks et al., 2016). However, the lack of support for NMJ disruption in the current study, discussed below, suggests that relative preservation of fibre size is the primary reason for the preserved MUP area in the Exercise group.

MUFR decreased following bedrest, which is consistent with previous reports observed when contractions are normalized both to post‐disuse strength (Sarto et al., 2022; Valli et al., 2023) and to baseline values (Inns et al., 2022). The decrease was observed only in the Exercise group, whereas MUFR remained essentially unchanged in Controls. Since the Control group experienced a significantly larger decline in maximal strength (∼19%) compared to the Exercise group (∼8%), the Control participants were effectively contracting at a slightly higher relative percentage of their new maximum capacity (∼31% vs. ∼27%) during the post‐test. This elevated relative effort in the Control group likely necessitated the maintenance of higher discharge rates to generate the required force. This effectively masked the physiological decrease in MUFR, or muscle wisdom, that would be expected with disuse‐induced slowing of contractile properties. Once recruited, the rate at which a MU discharges action potentials regulates muscle force production, with higher MUFR required to generate larger forces (Enoka & Duchateau, 2017). When viewed alongside the probable preservation of muscle fibre cross sectional area in the Exercise group, the required MUFR to achieve these target forces was lower in the Exercise group, which would help to explain the reduction of MUFR in the Exercise group. These findings underscore that neural and muscular components can be affected differently by disuse and countermeasures. The effective preservation of strength in the Exercise group appears to result from the maintenance of the muscular component (preserved MUP area and quadriceps volume) occurring alongside a physiological optimization of the neural component.

Exploration of peripheral electrophysiological measures showed minimal change in both groups following HDBR. The negative peak ratio did not differ from pre to post, with no difference in de‐ and repolarization profiles of individual MUs. Similarly, although CAF has been suggested as a blood‐based biomarker for NMJ structure and/or function, with associative data in human disuse studies (Monti et al., 2021; Sarto et al., 2022), plasma CAF showed no significant change in the current study. This was supported by electrophysiological findings, showing no adaptation of NMJ transmission instability in either group. These latter findings contrast with those recently reported by Motanova et al. in older men (68.5 ± 2.64 years) following 10 days of bedrest using similar methods (Motanova et al., 2025). This discrepancy may reflect the high interindividual variability inherent in a mixed‐sex cohort, compounded by the simultaneous assessment of bed rest and exercise effects in a relatively small sample of 23 participants who completed both the pre‐bedrest (baseline) and 14‐day head‐down tilt phases, of whom 15 completed the necessary assessments for our study.

Finally, it is important to acknowledge that the neural drive generated during submaximal isometric tasks is considerably lower than that required for explosive contractions. As noted in recent reviews, disuse‐induced neurophysiological impairments, such as intrinsic motoneuron remodelling, are often most pronounced during tasks requiring rapid force development (Ruggiero & Gruber, 2024). Although explosive strength assessments are often included in bedrest protocols, acquiring stable MU recordings during such rapid contractions presents significant technical challenges. Consequently, our analysis was limited to submaximal isometric efforts. Future research should aim to couple explosive contraction protocols with advanced electromyographic techniques to capture the full spectrum of high‐threshold MU adaptations following bedrest.

### 4.1 Study limitations and future directions

One limitation of this study is the relatively short duration of bedrest, which may not fully capture the longer‐term effects of disuse on neuromuscular properties. Additionally, although the study was initially powered to detect significant effects in neuromotor drive with 24 participants (12 per group), only 23 completed the bed rest, and 15 provided complete data for the present analyses. This reduced sample size likely limited the statistical power for some outcomes and may affect the generalizability of our findings. Furthermore, while our cohort included both men and women, this limited final sample size precluded a robust statistical assessment of sex‐specific effects. Future studies with larger cohorts and longer bed rest periods are warranted to replicate and extend these results. Regarding the translational relevance of our findings, we acknowledge that the intensive exercise protocol used in this study may not be immediately applicable to all real‐world clinical scenarios. Patients bedridden due to acute pathology or severe frailty may initially be unable to perform such high‐intensity countermeasures. Furthermore, the implementation of such a comprehensive, supervised programme requires significant personnel and time resources, which may be challenging to replicate in standard or resource‐limited clinical care settings. However, establishing the physiological efficacy of these interventions in a controlled HDBR model is a critical prerequisite for developing adapted, more feasible rehabilitation strategies for these vulnerable populations. Also, it is important to contextualize the physiological targets of our countermeasure. While the exercise protocol attenuated the loss of maximal isometric strength, it did not specifically target explosive strength capabilities. Disuse elicits a disproportionately greater decline in the rate of force development compared to maximal force, primarily due to impairments in rapid neural activation and intrinsic MU properties (Ruggiero & Gruber, 2024). Standard resistance training, while effective for force maintenance, lacks the high strain rates necessary to counteract these rapid‐contractile deficits.

Future research should explore the effects of different types and intensities of exercise countermeasures, such as plyometrics or resistive vibration exercise, on neuromuscular function during bedrest and within healthcare settings to extend the findings to patient groups such as older adults experiencing hospitalization, immobilization or chronic inactivity. Investigating the underlying mechanisms of MU adaptations in older adults, patient groups and with spaceflight will be crucial for developing targeted interventions.

Our findings offer the first insights into the effects of bedrest on MU properties in older males and females and have important implications for clinical settings and for human spaceflight. The ability to maintain muscle health and function during periods of inactivity is critical for the well‐being of clinical populations and astronauts. Consequently, our findings underscore the importance of developing effective exercise countermeasures to mitigate the adverse effects of disuse and support healthy ageing.

### 4.2 Conclusion

This study provides novel insights into the neuromuscular effects of HDBR in older males and females. The findings demonstrate that exercise attenuated declines in strength and MUP area, suggesting preservation of muscle fibre integrity and recruitment properties despite a small overall loss in leg lean mass. In contrast, the Control group exhibited declines in both knee extensor strength and MUP area, with little change in MUFR. Moreover, the lack of changes in circulating CAF, together with the preserved stability of NMJ transmission, suggests that HDBR has minimal impact on the NMJ in older adults. These findings highlight the efficacy of exercise interventions in mitigating disuse‐induced muscle weakness and may have broader implications for other conditions characterized by reduced physical activity.

## AUTHOR CONTRIBUTIONS

The bedrest study was conducted at the McGill University Health Centre, and biochemical blood analyses were performed at the Research Centre on Aging of the Université de Sherbrooke. Study conception: Isabelle J. Dionne, Eleonor Riesco, Jamie S. McPhee and Ahmed Ghachem; Data acquisition: Philippe St‐Martin, Jean‐Christophe Lagacé and Mathil Ruel; Data analysis: Philippe St‐Martin and Mathew Piasecki; Manuscript drafting: Philippe St‐Martin, Mathew Piasecki and Jamie S. McPhee; Critical manuscript revision: All authors. All authors approved the final version of the manuscript and agree to be accountable for all aspects of the work in ensuring that questions related to the accuracy or integrity of any part of the work are appropriately investigated and resolved. All persons designated as authors qualify for authorship, and all those who qualify for authorship are listed.

## CONFLICT OF INTEREST

The authors declare that they have no conflicts of interest.

## Data Availability

Due to ethical restrictions and legal requirements regarding the privacy of human participants in this bedrest study, the data are not publicly available. Sharing the raw data would compromise the ethical standards agreed upon in the participant consent forms. However, anonymized data are available from the corresponding author upon reasonable request.
